# Mapping the brain’s fatigue network: a transdiagnostic systematic review and meta-analysis on functional correlates of mental fatigue

**DOI:** 10.1093/braincomms/fcaf315

**Published:** 2025-08-28

**Authors:** Andy Schumann, Monica Di Giuliano, Steffen Schulz, Feliberto de la Cruz, Teresa Kreuder, Georg Seifert, Karl-Jürgen Bär

**Affiliations:** Lab for Autonomic Neuroscience, Imaging and Cognition (LANIC), Department of Psychosomatic Medicine and Psychotherapy, Jena University Hospital, Krautgasse 8, 07743 Jena, Germany; Lab for Autonomic Neuroscience, Imaging and Cognition (LANIC), Department of Psychosomatic Medicine and Psychotherapy, Jena University Hospital, Krautgasse 8, 07743 Jena, Germany; Charité Competence Center for Traditional and Integrative Medicine (CCCTIM), Charité—Universitätsmedizin Berlin, Corporate Member of Freie Universität Berlin, Humboldt-Universität zu Berlin and Berlin Institute of Health, Augustenburger Platz 1, 13353 Berlin, Germany; Lab for Autonomic Neuroscience, Imaging and Cognition (LANIC), Department of Psychosomatic Medicine and Psychotherapy, Jena University Hospital, Krautgasse 8, 07743 Jena, Germany; Lab for Autonomic Neuroscience, Imaging and Cognition (LANIC), Department of Psychosomatic Medicine and Psychotherapy, Jena University Hospital, Krautgasse 8, 07743 Jena, Germany; Charité Competence Center for Traditional and Integrative Medicine (CCCTIM), Charité—Universitätsmedizin Berlin, Corporate Member of Freie Universität Berlin, Humboldt-Universität zu Berlin and Berlin Institute of Health, Augustenburger Platz 1, 13353 Berlin, Germany; Lab for Autonomic Neuroscience, Imaging and Cognition (LANIC), Department of Psychosomatic Medicine and Psychotherapy, Jena University Hospital, Krautgasse 8, 07743 Jena, Germany

**Keywords:** arterial spin labeling, functional magnetic resonance, positron emission tomography, fatigability, mental disorders

## Abstract

Mental fatigue is a significant psychopathological symptom that has recently gained attention, particularly in chronic fatigue syndrome/myalgic encephalomyelitis and Post–COVID-19 condition. However, fatigue is a clinically relevant symptom across a wide range of mental and neurological disorders. To identify a transdiagnostic functional network associated with fatigue, we conducted an activation likelihood estimation meta-analysis of neuroimaging studies. The primary inclusion criterion was studies involving any medical condition where patients exhibited significantly higher levels of fatigue compared to healthy controls. A systematic literature review across three major scientific databases identified 46 eligible neuroimaging studies, including a total of 2603 individuals. The meta-analysis of these studies revealed a widespread cortical–subcortical network involving frontal, limbic, basal ganglia and parietal structures. Three main clusters were highlighted: a frontal–striatal–limbic cluster, a frontal–cingulate cluster and a parietal cluster, with regions implicated in cognitive, emotional and somatosensory symptoms associated with mental fatigue. Quality analysis indicated a moderate risk of bias in the majority of the included studies. Overall, our findings provide scientific evidence for a transdiagnostic mental fatigue network in the brain, with key nodes located in the lateral frontal cortex, cingulate cortex, insula, thalamus, precuneus and caudate. These results support the theory of thalamic–striatal–cortical dysfunction, which may impair compensatory mechanisms related to mental fatigue. Additionally, abnormal activation of limbic and parietal regions may contribute to cognitive, emotional and attentional impairments linked to fatigue.

## Teaser

Schumann and Di Giuliano *et al*. conducted a meta-analysis of 46 neuroimaging studies with 2603 individuals to identify a brain network linked to mental fatigue across disorders. They found consistent activation in frontal–striatal–limbic, frontal–cingulate, and parietal clusters, which appear to be key correlates of mental fatigue.

## Graphical Abstract

**Graphical Abstract:**
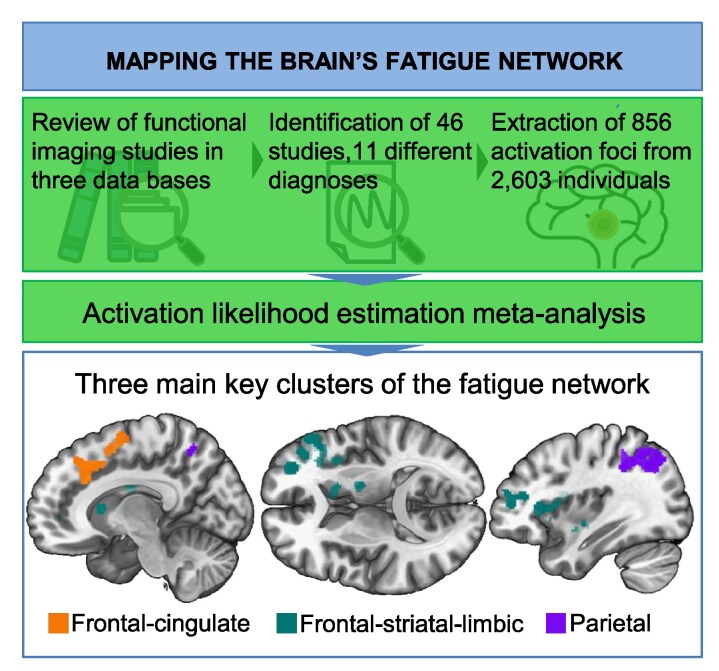

## Introduction

What happens when the body's natural need to conserve energy shifts to the extreme, where ‘fatigue’ becomes a key health issue? Common difficulties in concentration, weakness, dyspnoea, somnolence or low mood are typically associated with this construct.^1,2^ Fatigue may be classified as physiologic or pathologic, where the pervasiveness and persistence of its correlated symptoms allow to differentiate between these two macro-categories.^3^ Physiologic fatigue implicates post-prandial, post-sleep, pre-sleep, post-exercise and physical deconditioning. Pathological fatigue can be classified as characterized by mental and physical dimensions to take into consideration: mental fatigue refers to specific psychological and psychiatric conditions; physical fatigue relates to neurologic (primary) or non-neurologic (secondary) manifestations. Specifically, neurological manifestation of fatigue includes central or peripheral system pathophysiological mechanisms. On the other hand, non-neurological manifestation refers to immunological, rheumatological, haematological, endocrinological and drugs/irradiation/intoxication effects.^4,5^ Moreover, fatigue can occur at rest or during a specific physical/mental task performance, in acute or chronic manifestation (>6 months). A key difference should be accounted also according to a subjective sense (perceived fatigue) or an objective sense of fatigue, that is a measurable phenomenon of fatigability.^5,6^

The cognitive and physical symptoms associated with fatigue are commonly frequent in many disorders. Specifically, fatigue is highly prevalent in neuropsychiatric disorders, inflammatory-rheumatic diseases, cancer, myalgic encephalomyelitis and Fibromyalgia Syndrome.^7-11^ In these disorders, fatigue strongly impacts quality of life.^8,9^ Moreover, when fatigue persists for longer than 6 months and is present more than 50% of the time, accompanied by significant functional impairment and psychological distress, this leads to a diagnosis of chronic fatigue syndrome (CFS).^12-14^ Chronic fatigue individuals exhibit a wide range of neuropsychological and medical disorder-defining traits: short-term memory impairments, sore throat, tender lymph nodes, muscular/joint pain, headache, sleep problems and post-exertional malaise.^15^ Whereas individuals with CFS should meet at least four of the six listed neuropsychological and medical symptoms for a diagnosis, it is worth to outline that there are conditions in which individuals report or show a sustained fatigue in complete absence of underlying known medical conditions: this condition is better referred as ‘idiopathic chronic fatigue’. This condition is speculated to be related to age-related metabolic changes that could exacerbate fatigue symptoms expression.^12,15,16^

Fatigue is one of the most current and insidious psychosocial and physical side-effects of post-infectious syndromes, like Long COVID.^17,18^ Persistent fatigue is reported by a significant portion of patients affected by COVID, at 16–20 weeks post-symptom onset: it has been established that Post–COVID-19 condition or Long COVID is characterized by a state of chronic fatigue as severe as in other fatigue-related disorders.^2,19,20^ In this context, different fatigue brain-related activations have been discussed for Post–COVID-19 patients: persistent cognitive fatigue has been associated with prefrontal, basal ganglia and insular activations sustaining greater mental fatigue during effort-reward related tasks (e.g. *N*-back task). The greater self-reported effort during these kinds of tasks goes along with impaired motivational/interoceptive processes characterizing the whole neuropsychological profile of patients affected by SARS-CoV-2.^21^

Among the endophenotypic candidates which could explain fatigue, the most commonly discussed are: inflammatory processes, stress and related disturbances in the hypothalamic–pituitary–adrenal axis, immune-mediated dopaminergic (DA) and serotonergic signalling changes.^7-10,17,18,22,23^ Incidentally, fatigue can be caused by a central nervous system dysfunction, involving a chain reaction from the body’s receptors, through the spinal cord, and specific functional areas of the brain, to the terminal mitochondria.^24^

Adopting a transdiagnostic perspective on fatigue could enhance the understanding of various range of psychopathological conditions and the implementation of effective interventions: this approach highlight the relevance of symptom-dependency and disease-independency, outlining common underlying prognostic and maintenance factors that contribute to fatigue.^25,26^ Given these premises, deepening the multidimensionality of the construct is necessary for both assessments and interventions.^25^ In this context, a constant and ongoing discussion on the definition and nature of the construct is still widely present in literature.^27^ As a matter of fact, there is no standard way of measuring fatigue: whether the measurements implemented are subjective (self-reports/questionnaires) or objective (standardized tasks), the dimensionality of the construct and its assessment is a timely open issue.^25,27^

Along with perception, cognition and emotions, fatigue could be translated in a specific brain network architecture.^1,8,22,23,28^ Fatigue has been commonly associated with activation of ventral striatal subcortical areas, the ventro-medial prefrontal cortex (vmPFC), the dorsolateral prefrontal cortex (DLPFC) and limbic structures as the anterior insula and dorsal anterior cingulate cortex (dACC). Current reviews in the healthy population converge with these findings, pinpointing the role of limbic, striatal and prefrontal areas in sustaining the motivational-reward fatigue neuronal stream.^29-31^ It has been discussed how functional connectivity between these regions and other frontal regions largely decreased along with cognitive fatigue increment, while connectivity between these seeds and more posterior regions increased: these findings pose the striatum, the DLPFC, the insula and the vmPFC appeared to be central ‘nodes’ or hubs of the fatigue network.^30^ Specifically, these cortical–subcortical areas are relevant as far as interoceptive awareness and decision-making/reward approaches related to fatigue during task performances are concerned.^30,32^ During resting state conditions, connectivity disruptions between cortical regions of the Default Mode Network (DMN) and several brain regions have been observed in CFS conditions, which have a strong correlation with depression and fatigue symptomatology.^33-35^ On the other hand, in healthy population, reduced anti-correlations between regions of the DMN, the posterior cingulate and the prefrontal cortex have been detected following prolonged mental workload, which may suggest that the increased intrinsic functional connectivity within this network may be associated with mental fatigue.^35^ The overall functional connectivity patterns found in CFS and healthy controls (HCs) build the basis for shared fatigue neuronal correlates in both groups.^35^ The assessment of fatigability can be further clusterized in different domains.^3,5,6,36-40^ Thus, given the significance of fatigue as a pervasive and persistent psychiatric symptom across various illnesses, a systematic meta-analysis of its brain functional correlates is timely and necessary. This could allow us to uncover the transdiagnostic cortical–subcortical mechanisms which sustain fatigue. We chose the meta-analytic activation likelihood estimation (ALE) method to evaluate commonly activated regions across multiple studies.^41^ We chose to focus on neuroimaging studies which have provided a systematic evaluation of fatigue network in clinical samples with a direct comparison to HCs, taking into account different factors which could contribute to build the fundamentals of our cutting-edge perspective on fatigue, in a transdiagnostic way. We include all the possible neuroimaging studies which have assessed fatigue according to different perception, motor, cognitive and affective domains facet of the construct, in task-design approaches; on the other hand, we included also resting state imaging studies which shed a light on common and differential fatigue–brain connectivity pathways. Overall, we aim to highlight which are the relevant cortical–subcortical hubs of mental fatigue at rest and during fatigue-dimension design, in order to achieve a current state of art of this transdiagnostic trait.

## Materials and methods

### Study selection

To investigate the transdiagnostic characteristics of a brain network related to fatigue, we followed the strategy outlined in the pre-registration record CRD42023414657^42^ in the International Prospective Register of Systematic Reviews (PROSPERO) (https://www.crd.york.ac.uk/prospero). As of 1 June 2024, three different databases—PubMed/MEDLINE (www.PubMed.ncbi.nlm.nih.gov), Embase (www.embase.com) and the APA PsycInfo database (http://www.apa.org/psycinfo)—were screened for relevant publications. The search text was ‘(“arterial spin labeling” OR “ASL” OR “functional magnetic resonance” OR “fMRI” OR “positron emission tomography” OR “PET” OR “single-photon emission computed tomography” OR “SPECT”) AND [(“fatigue” OR “fatigability” OR “post exertional malaise” OR “post-exertional malaise”) AND (“cognitive” OR “mental” OR “central”)]’.

One of the main eligibility criteria was to include studies with any medical condition as long as their fatigue levels were significantly increased in the sample under investigation, in comparison to HCs. In this regard, collected articles were selected according to the following inclusion criteria: (i) quantitative assessment of fatigue, including subjective measures (e.g. self-report like Visual Analog Scale for Fatigue or Chalder Fatigue Scale); (ii) significantly elevated level of fatigue in the clinical sample compared to HCs; (iii) diagnosis of the medical condition in accordance to recognized diagnostic criteria; (iv) reported standard space coordinates of the brain regions (Montreal Neurological Institute or Talairach); (v) peer-reviewed publication; (vi) original research articles, in English. Search filters were used when available to include only articles in English language involving humans.

Exclusion criteria encompass: conceptual frameworks, viewpoints, case reports, meta-analyses, reviews, study protocols, non-peer-reviewed studies (preprints, theses), studies not in English and studies without an abstract. The eligibility SPIDER criteria were stated as follows^43^: the sample (S) includes patients who experience fatigue, the phenomenon of interest (PI) focuses on brain function related to mental fatigue, the design (D) is observational, including cohort and case–control studies but excluding single case studies, the evaluation (E) involves spatial coordinates of differences in brain function (comparison patients versus controls) or coordinates of brain functional measures correlated with fatigue scores, and the research type (R) involves quantitative analysis of functional brain imaging data.

**Figure 1:**
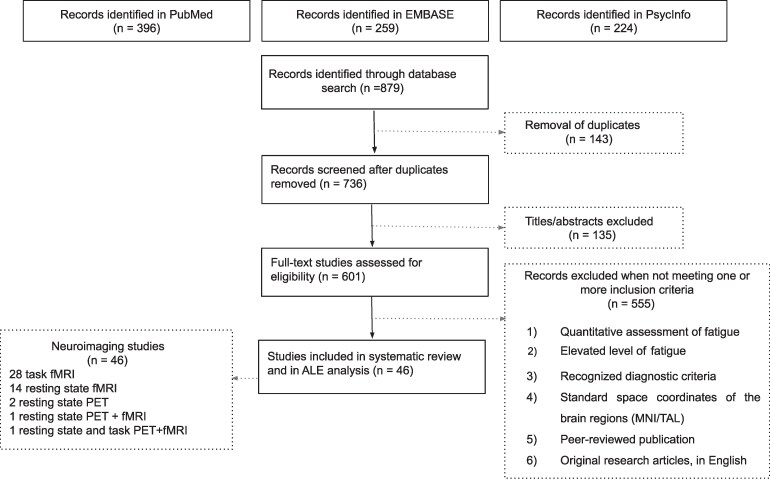
**PRISMA flowchart process of article selection according to PRISMA guidelines.** ALE, activation likelihood estimation; fMRI, functional magnetic resonance imaging; PET, positron emission tomography; MNI, Montreal Neurological Institute; TAL, Talairach; PRISMA, Preferred Reporting Items for Systematic Reviews and Meta-Analyses.

The research strategy and study selection can be visualized in terms of the Preferred Reporting Items for Systematic Reviews and Meta-Analyses (PRISMA) flowchart (Fig. 1).

### Procedure and studies classification

Two independent reviewers have screened all hits from the literature search and selected eligible studies for inclusion in the systematic review according to the specified criteria. The two reviewers were blinded to each other’s decisions. A third researcher resolved disagreements between individual judgments. Coordinates of brain regions (foci) were extracted together with study details (i.e. number of subjects, diagnosis of clinical population, fatigue assessment, imaging technique, type of experimental design and neuropsychological dimension investigated in case of task designs). Specifically, we first looked for a pool analysis of all the studies and then we analysed studies on different diagnoses represented by a pre-defined minimum number of three studies available for an individual meta-analysis [Gulf War Illness (GWI), multiple sclerosis (MS), Parkinson disease (PD), traumatic brain injury (TBI) and CFS].

### Analysis of influencing factors

To investigate more specifically some factors potentially influencing our results we assessed some key characteristics from the eligible studies. Regarding the type of task dimension, have categorized the neuroimaging task designs according to three main neuropsychological dimension: cognitive (e.g. alertness task; *n*-Back task), emotional (e.g. emotional faces reactions task) and sensorimotor (e.g. imagery tasks, finger tapping).

Additionally, we extracted the means and standard deviations of fatigue ratings from all eligible studies to estimate Cohen’s *d* effect sizes.^44^ When means and standard deviations were not reported, we approximated them using the median and range.^45^ If summary statistics (e.g. *t*- or *F*-values) were reported instead of group distributions, effect sizes were estimated directly from these statistics.^46^

We used Newcastle–Ottawa scale (NOS) for assessing the quality of each of the eligible studies to evaluate a potential risk of bias.^47^ The NOS can be applied to rate non-randomized studies such as case–control in literature reviews or meta-analyses. Eligible studies were analysed by two researchers, independently. Conflicts were resolved by consulting a third researcher.

NOS has nine items covering selection of case and controls (four), comparability of cases and controls (two), and ascertainment of exposure (three). In this analysis, we did not generally consider right-handedness as a selection bias. Furthermore, we assumed that all participants responded unless information about non-responders was provided. Controlling variables were identified as covariates in the statistical analysis of brain imaging data (e.g. in a second-level ANCOVA).

### Statistical analysis

We assessed transdiagnostic and diagnosis-specific maps of brain regions associated with cognitive fatigue by coordinate-based meta-analyses.^48,49^ The random-effects approach of the ALE meta-analysis was conducted using the GingerALE software version 3.0.2 (http://www.brainmap.org/ale). Coordinates in Talairach space were transformed into Montreal Neurological Institute (MNI) space.

When a specific statistical analysis in an article revealed a set of brain regions with different functional behaviour in patients with fatigue compared to HCs (e.g. increased activation during a task), their peak coordinates were included in the meta-analysis as a group of foci. Each of these foci were weighted by the number of subjects included in that specific analysis to create so-called modelled activation maps. The ALE approach computes the statistical spatial convergence of the activation maps across independent analyses and studies. Using a permutation procedure, the estimate of true convergence of foci was tested against the null hypothesis of random spatial distribution of foci between experiments.^50^

Resulting ALE maps were thresholded (*P* < 0.05; 1000 permutations, *P* < 0.05 cluster forming threshold, FWE corrected) and registered to the MNI152 template. To assess brain regions related to fatigue commonly reported across diagnoses, we conducted an ALE analysis of all studies deemed eligible in the systematic literature review. Then, we look at the different and overlapping functional activations across all the disorder categories with a minimum number of three studies (GWI, MS, PD, TBI and CFS). As the number of studies and foci are comparably low, we did not use FWE-correction on cluster-level and used a minimum cluster size of 1000 voxels, instead. The condition-specific activation maps were compared qualitatively (spatial overlap between maps).

From ALE results of the pooled analysis, we traced back the individual studies that contributed foci to each of the statistically significant clusters. After grouping these studies by clinical context, we then assessed the contribution of diagnostic groups to the clusters (see Tables 1 and 2).

**Table 1:** Characteristics of task design studies

Authors	Diagnosis	Samples (*n*)	Assessment	Task design	Dimension
Amann *et al*. (2011)^51^	MS	MS = 16, HC = 16	FSMC, MFIS	Alertness task, *n*-Back task	Cognitive
Arwert *et al*. (2006)^52^	GHD	GHD = 13, HC = 13	POMS	DNMTS task	Cognitive
Berginström *et al*. (2018)^53^	TBI	TBI = 57, HC = 27	FSS	mSDMT task	Cognitive
Boissoneault *et al*. (2018)^54^	CFS	CFS = 19, HC = 15	VAS-F	PASAT	Cognitive
Bruijel *et al*. (2022)^55^	TBI	TBI = 16, HC = 17	FSS, VAS-F	*n-back*	Cognitive
Cagna *et al*. (2023)^56^	MS	MS = 29, HC = 25	FSS	Feedback-based, paired-associative word learning task	Cognitive
Caseras *et al*. (2006)^57^	CFS	CFS = 17, HC = 12	ChFS	*n*-back task	Cognitive
Caseras *et al*. (2008)^58^	CFS	CFS = 12, HC = 11	ChFS	Fatigue, relaxation and anxiety provocation task	Cognitive, Emotional and sensorimotor
Chen *et al*. (2020)^59^	MS	MS = 19, HC = 17	VAS-F	msDMT	Cognitive
Cook *et al*. (2007)^60^	CFS	CFS = 9, HC = 11	POMS	PASAT	Cognitive
Cook *et al*. (2017)^61^	CFS	CFS = 15, HC = 15	POMS, VAS-F	PASAT, simple number recognition and finger tapping	Cognitive, sensorimotor
De Lange *et al*. (2004)^62^	CFS	CFS = 16, HC = 16	CIS-R	MI and VI tasks	Sensorimotor
Dobryakova *et al*. (2020)^63^	TBI	TBI = 21, HC = 24	MFIS, VAS-F	Modified card-guessing task	Cognitive
Genova *et al*. (2013)^64^	MS	MS = 12, HC = 11	VAS-F	Task-switching paradigm	Cognitive
Glass *et al*. (2011)^65^	FM	FM = 18, HC = 14	MFI	Go/No-Go	Cognitive
Liu *et al*. (2016)^66^	TBI	TBI = 25, HC = 20	FAI	PVT	Cognitive
Menning *et al*. (2017)^67^	BC	BC = 52, HC = 31	QLQ-C30	Verbal recall task	Cognitive
Mizuno *et al*. (2015)^68^	CFS	CFS = 15, HC = 13	ChFS	Modified version of the kana Pick-out test-KPT	Cognitive
Nordin *et al*. (2016)^69^	TBI	TBI = 10, HC = 10	FSS	PVT	Cognitive
Provenzano *et al*. (2020)^70^	GWI	GWI = 80, HC = 31	CMI	*n*-back	Cognitive
Spiteri *et al*. (2019)^71^	MS	MS = 40, HC = 22	VAS-F	*n*-back	Cognitive
Svolgaard *et al*. (2018)^72^	MS	MS = 44, HC = 25	FSMC	Tonic grip-force task	Sensorimotor
Svolgaard *et al*. (2022)^73^	MS	MS = 44, HC = 22	FSMC	Tonic grip-force task	Sensorimotor
Wagner *et al*. (2022)^74^	MS	MS = 18, HC = 15	FSS	ANT task	Cognitive
Washington *et al*. (2020)^75^	GWI	GWI = 80, HC = 31	ChFS	*n* back	Cognitive
Washington *et al*. (2020)^76^	GWI + CFS	GWI = 80, CFS = 38, HC = 31	ChFS	*n* back	Cognitive
Wortinger *et al*. (2017)^77^	CFS	CFS = 15, HC = 24	ChFS	Emotional conflict task	Emotional
Zhao *et al*. (2024)^78^	PC	PC = 52, HC = 35	FS-14	*n*-back	Cognitive
Zunini *et al*. (2013)^79^	BC	BC = 21, HC = 21	POMS	Tol, paired associates memory task	Cognitive

Nomenclature of the disorders: CFS = Chronic fatigue syndrome/myalgic encephalitis; GHD = Growth hormone deficiency; MS = Multiple sclerosis; BC = Breast cancer; PD = Parkinson disease; TBI = Traumatic brain injury; GWI = Gulf war illness; PC = Post–COVID-19 condition. List of reported questionnaires: CIS-R = Checklist Individual Strength Revised; POMS = Profile of Mood State questionnaires; ChFS = Chalder Fatigue Scale; FSS = Fatigue Severity Scale; FSMC = Fatigue Scale for Motor and Cognitive Functions; MFIS = Modified Fatigue Impact Scale; VAS-F = Visual Analog Scale for Fatigue; MFIS = Multidimensional Fatigue Inventory scale; FAI = Fatigue Assessment Index; QLQ-C30 = European Organization for Research and Treatment of Cancer health-related Quality-of-life Questionnaire; ACR = American College of Rheumatology research classification criteria; CMI = Chronic Multisymptom Illness; FS-14 = Fatigue Scale-14. List of reported task design, according to the main neuropsychological dimension investigated: **cognitive** = alertness task; *n-*Back task; delayed-non-matching-to-sample task (DNMTS); Symbol Digit Modalities Test (msDMT); the Paced Auditory Serial Addition Test (PASAT); a feedback based, paired-associative word learning task; simple number recognition task; modified card-guessing task (monetary reward task); task-switching paradigm; Go/No-Go task; psychomotor vigilance test (PVT); Tower of London task (ToL); kana Pick-out Test; attention network task (ANT); **emotional** = emotion conflict task (emotional faces reactions); **sensorimotor** = finger tapping; tonic grip force task; motor (MI) and visual (VI) imagery tasks.

**Table 2:** Characteristics of resting state design studies

Authors	Diagnosis	Sample (*n*)	Assessment	Modality
Alshelh *et al*. (2020)^80^	GWI	GWI = 15, HC = 33	ACR	PET
Boissoneault *et al*. (2016)^81^	CFS	CFS = 17, HC = 17	VAS-F	fMRI
Bruijel *et al*. (2022)^55^	TBI	TBI = 16, HC = 17	FSS, VAS-F	fMRI
Fallon *et al*. (2009)^82^	LD	LD = 35, HC = 17	FSS	PET
Gay *et al*. (2016)^34^	CFS	CFS = 19, HC = 17	MFIS	fMRI
Guo *et al*. (2023)^83^	ID	ID = 42, HC = 22	FSS	fMRI
Herren *et al*. (2011)^84^	HCV	HCV = 15, HC = 16	FIS	PET
Hesse *et al*. (2014)^85^	MS	MS = 23, HC = 22	WEIMuS	PET
Li *et al*. (2017)^86^	PD	PD = 49 (17 PD-F and 32 PD-NF), HC = 25	FSS	fMRI
Lin *et al*. (2019)^87^	MS	MS = 64, HC = 26	FSS	fMRI
Shan *et al*. (2023)^88^	PD	PD = 38, HC = 23	FSS	fMRI
Staud *et al*. (2018)^89^	CFS	CFS = 17, HC = 16	VAS-F	fMRI
Wang *et al*. (2021)^90^	TBI	MS = 42, HC = 62	CMI	fMRI
Wortinger *et al*. (2016)^91^	CFS	CFS = 18, HC = 18	ChFS	fMRI
Wortinger *et al*. (2017)^92^	CFS	CFS = 18, HC = 18	ChFS	fMRI
Wu *et al*. (2016)^93^	MS	MS = 22, HC = 22	MFIS	fMRI
Zhang *et al*. (2017)^94^	PD	PD = 49, HC = 25	FSS	fMRI
Zhou *et al*. (2016)^95^	MS	MS = 34, HC = 34	MFIS	fMRI

Nomenclature of the disorders: CFS = Chronic fatigue syndrome/myalgic encephalitis; MS = Multiple sclerosis; HCV = Hepatitis C virus infection; LD = Lyme disease; PD = Parkinson disease; TBI = Traumatic brain injury; GWI = Gulf war illness; ID = Insomnia disorder; List of reported questionnaires: ACR = American College of Rheumatology research classification criteria; ChFS = Chalder Fatigue Scale; FSS = Fatigue Severity Scale; MFIS = Modified Fatigue Impact Scale; FIS = Fatigue Impact Scale; VAS-F = Visual Analog Scale for Fatigue; WEIMuS = Würzburg Fatigue Inventory in Multiple Sclerosis; MFIS = Multidimensional Fatigue Inventory Scale; CMI = Chronic Multisymptom Illness.

Brain maps were created in Surf Ice and MRIcroGL (McCausland Center for Brain Imaging, University of South Carolina). The contributions to each cluster were illustrated by cake plots (Excel 2019, Microsoft Corporation) while contributing foci were visualized by a bar plot (matplotlib, python 3.11).

## Results

Our database search for studies on fatigue-related brain networks yielded a total of 438 articles. After applying the eligibility criteria, 46 neuroimaging studies were included in the final analysis. These comprised 29 task-based fMRI studies and 18 resting-state studies using either PET or fMRI (one study reported both resting-state and task-based results; see Tables 1 and 2). Across all studies, a total of 2603 participants contributed 856 activation foci to the ALE analysis. Coordinates from each study were extracted and entered into the ALE analysis. The study selection process is summarized in Fig. 1, following the PRISMA guidelines.

**Figure 2:**
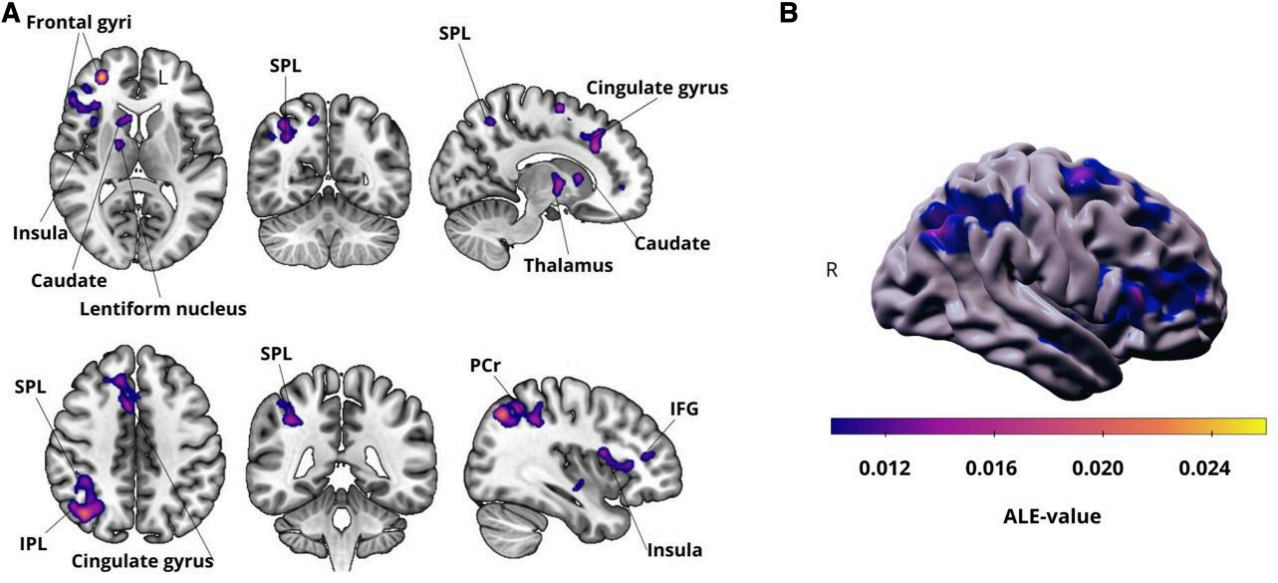
**Mental fatigue transdiagnostic network ALE analysis on the 46 eligible studies. A** Resulting ALE maps were thresholded (*P* < 0.05; 1000 permutations, *P* < 0.05 cluster forming threshold, FWE corrected) and registered to the MNI152 template. **B** The same results registered to Conte69 surface brain template. The pooled analysis of all studies (46 studies, 2603 subjects, 856 foci) revealed significant widespread cortical–subcortical activations characterizing the fatigue network. IFG, inferior frontal gyrus; SPL, right superior parietal lobule; IPL, inferior parietal lobule; PCr, right praecuneus.

The pooled analysis of all studies (46 studies, 2603 subjects, 856 foci) revealed significant widespread cortical–subcortical activations characterizing the fatigue network, consisting of three spatially distinct clusters. The frontal–striatal–limbic cluster covered regions in the medial, superior and middle frontal as well as the anterior cingulate gyrus, caudate nucleus, lentiform nucleus, thalamus, claustrum and insula. The frontal–cingulate cluster included the middle cingulate gyrus and supplementary motor area. The parietal clusters covered regions in the superior and inferior parietal lobule; angular gyrus, and praecuneus (see Fig. 2, Table 3).

**Table 3:** ALE clusters of the pooled analysis of all studies

Anatomical region	Size (mm^3^)	L/R	Max ALE score	MNI coordinates
Cluster 1: Frontal-striatal-limbic cluster				
Superior frontal gyrus	14 528	R/L	0.0175/0.0208	30 50 8/2 18 48
Middle frontal gyrus	14 528	R	0.0218	50 38 16
Inferior frontal gyrus	14 528	R	0.0010	46 24 4
Caudate	14 528	R	0.0223	20 -8 20
Lentiform nucleus	14 528	R	0.0242	18 12 0
Thalamus	14 528	R	0.0184	14 -2 2
Claustrum	14 528	R	0.0179	32 28 4
Insula	14 528	R	0.0164	38 6 16
Anterior cingulate gyrus	14 528	R/L	0.0152/0.0196	18 50 2/−6 32 22
Cluster 2: Frontal-cingulate cluster				
Medial frontal gyrus	11 648	R	0.0206	6 8 54
Cingulate gyrus	11 648	R/L	0.0184/0.0228	12 28 32/−10 24 36
Cluster 3: Parietal cluster				
Superior parietal lobule	8 992	R	0.0230	32 −68 50
Inferior parietal lobule	8 992	R	0.0181	48 −36 56
Angular gyrus	8 992	R	0.0162	46 −62 40
Praecuneus	8 992	R	0.0230	38 −68 44

There were 46 studies, 2603 subjects, 856 foci, 12.97 mm FWHM, and 1000 permutations. *P* < 0.05 is the cluster-forming threshold; *P* < 0.05 is the cluster threshold. L, Left; R, right.

Of note, for each of the three fatigue network clusters, we indicate information regarding the total number of studies, the type of experimental design (task versus resting state) and if present, the neuropsychological dimension investigated by each of the task studies (Table 4). We overall found that the majority of the studies are task-based in the experimental design, with only a small number of resting state studies contributing in the emerging fatigue network clusters. Nonetheless, a similar ratio in the distribution of both experimental designs can be observed in each cluster. Thus, none of the clusters is driven by a specific study design.

**Table 4:** Characteristics of the studies contributing to the three cluster

Mental fatigue network clusters	Total contributing studies	Task versus resting state studies	Task-dimension		
			Cognitive	Affective	Sensori-motor
Cluster 1: Frontal–striatal–limbic cluster	29/46	16 task and 8 resting state studies	14	1	2
Cluster 2: Frontal–cingulate cluster	23/46	11 task and 6 resting state studies	9	1	1
Cluster 3: Parietal cluster	15/46	10 task and 5 resting state studies	9	1	0

One of the studies contributing to cluster 1 implemented both a cognitive and a sensorimotor task to induce and assess fatigue [see Table 1, Cook *et al*. (2017)^61^]. That is why we classified this study also in the column referring to sensorimotor dimension.

**Figure 3:**
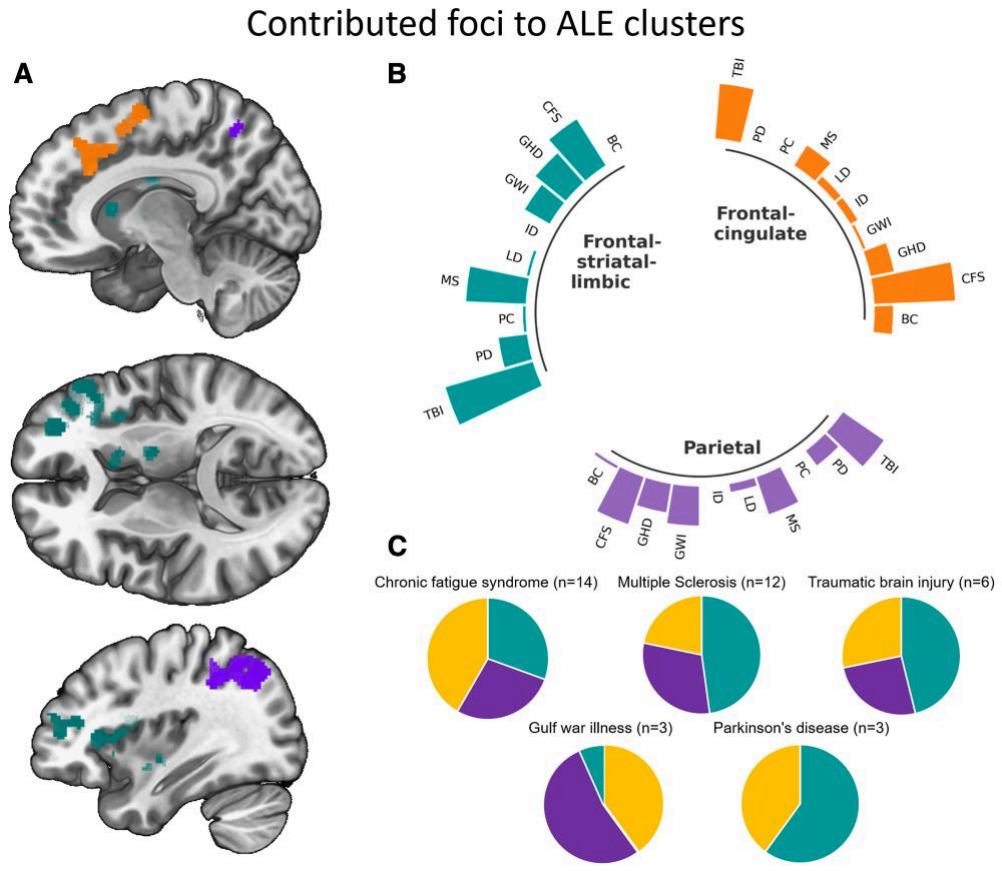
**Mental fatigue key transdiagnostic brain clusters contribution of foci to the three clusters (cyan: frontal–striatal–limbic cluster; orange: frontal–cingulate cluster; magenta: parietal cluster) revealed by meta-analysis of all studies. A** Spatial distribution of clusters in the frontal–striatal–limbic region, the frontal–cingulate region, and the parietal region. **B** Contribution of foci, grouped by diagnosis, to the three identified clusters. **C** Proportion of foci assigned to each cluster by diagnosis. All three clusters were present in every group except for PD that contributed to the frontal–striatal–limbic and the frontal-cingulate only. Abbreviations: BC, breast cancer; CFS, chronic fatigue syndrome/myalgic encephalitis; GHD, growth hormone deficiency; GWI, Gulf War illness; ID, insomnia disorder; LD, lyme disease; MS, multiple sclerosis; PC, Post–COVID-19 condition; PD, Parkinson’s disease; TBI, traumatic brain injury. See Table S2 to check the exact number of foci per each individual disorder-category contribution in elaborating the overall three clusters.

At the same time, we investigated the contribution of the studies on specific disorders to the overall foci pinpointed for each of the three clusters. The spatial extent of the frontal–striatal–limbic , the frontal–cingulate and the parietal cluster is shown in Fig. 3A. Their contributing foci were separated by the clinical context of the study (see Fig. 3B).

The diagnoses fibromyalgia and hepatitis C viral infection were both represented by one study only, that did not contribute any foci to the results. In the remaining 10 groups of disorders, studies had reported foci relevant to one or more clusters. Except for insomnia and breast cancer, every group contributed results to the frontal–striatal–limbic cluster with a majority of studies reporting multiple foci. At the same time, only Post–COVID-19 condition and PD for the frontal–cingulate cluster, along with insomnia and Post–COVID-19 condition for the parietal cluster, had not contributed to any of the foci to the results (Fig. 3B). In diagnostic groups with more than two studies, the proportion of foci contributed to each cluster is shown in Fig. 3C. The frontal–striatal–limbic and frontal–cingulate cluster were represented in all diagnoses. The parietal cluster was not in PD. Incidentally, depending on the aetiology of each disorder category, fine differential expression of each of these fatigue network clusters can be outlined (Fig. 3C).

**Figure 4:**
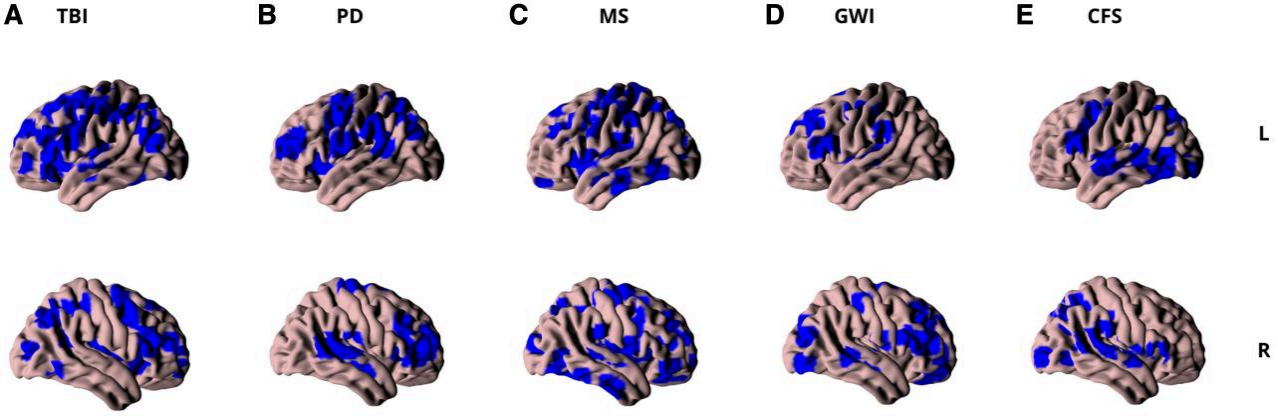
**Disease-specific facets of the fatigue network the individual analysis on different disorders contributing to a transdiagnostic fatigue network: A** traumatic brain injury (TBI, 6 studies, 343 subjects, 168 foci), **B** Parkinson's disease (PD, 3 studies, 210 subjects, 38 foci), **C** multiple sclerosis (MS, 12 studies, 585 subjects, 178 foci), **D** Gulf War illness (GWI, 3 studies, 270 subjects, 116 foci), **E** chronic fatigue syndrome (CFS, 14 studies, 630 subjects, 217 foci). All maps showed a widespread cortical–subcortical network with common and different functional brain activations according to the disease (resulting activation likelihood estimation maps were thresholded *P* < 0.05; 1000 permutations, *P* < 0.05 cluster forming threshold, minimum cluster size of 1000 voxels). See Supplementary Table S1 for specific location of each functional activations according to the diseases.

To further investigate consistencies and differences between disorder-specific brain maps associated with mental fatigue, individual meta-analyses were performed in each group with a minimum of three studies (GWI, MS, PD, TBI and CFS). We found areas related to the three main clusters of the pooled analysis (frontal–striatal–limbic, frontal–cingulate and parietal clusters) to be overall present across different disorders (Fig. 4). A comprehensive list of these findings (regions labelling, cluster size, hemispheric lateralization, ALE values and coordinates) in diagnosis-specific categories can be reviewed in the Supplementary materials (Table S1).

Risk of bias was assessed using the NOS. Ratings for all nine items are presented in Table S2 of the Supplementary Materials. The total risk of bias score is calculated by summing the number of stars, yielding scores that ranged from 3 to 8. Most studies were classified as having a moderate risk of bias, indicated by total scores between 4 and 6.

**Figure 5:**
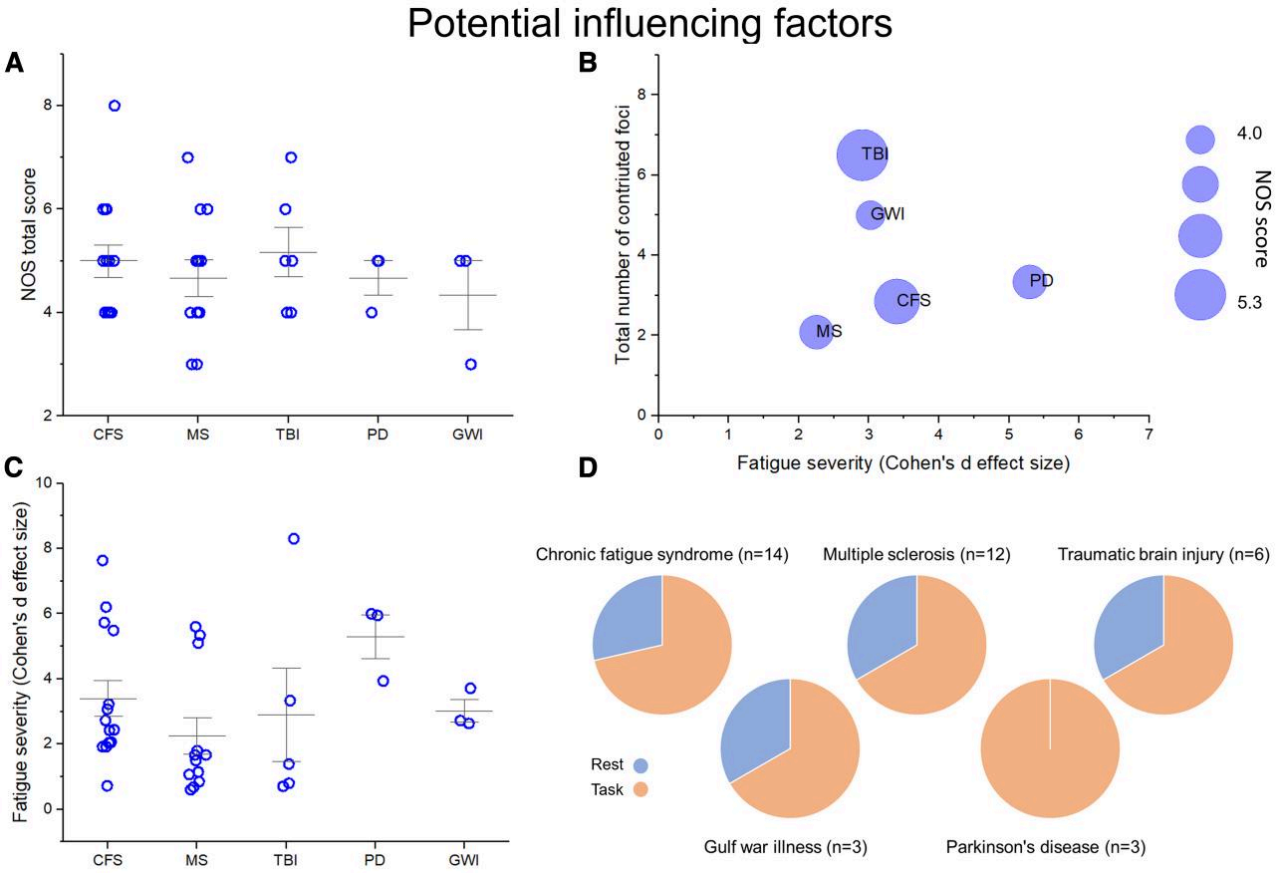
**Factors potentially influencing meta-analytic results. A** Mean study quality, assessed by the NOS score, across studies grouped by clinical sample. Each data point represents an individual study. **B** Relationship between mean fatigue severity and the number of foci contributed to the ALE results. Circle size indicates the mean NOS score. Each data point represents the average across all studies investigating a common clinical group. **C** Severity of fatigue reported by the studies within each diagnostic group. Each data point represents an individual study. **D** Proportion of task-based and resting-state neuroimaging designs across diagnostic groups. For PD, only task-based studies were identified. In all other groups, the majority of studies employed task-based designs.

Fatigue severity was quantified based on the statistical outcomes reported for fatigue scales in each study. To facilitate comparison, we approximated Cohen’s *d* effect sizes for group differences in fatigue scores between clinical populations and HCs, which ranged from 0.6 to 8.3. For the five most frequently represented diagnostic groups, we reported both fatigue severity and study quality in Fig. 5A–C. To assess their potential influence on our findings, we plotted the number of foci contributing to the ALE results against mean fatigue severity. In this visualization, the size of each circle represents the average study quality for each diagnostic group. The group of studies on TBI contributed the most foci and had the highest study quality, indicating the lowest risk of bias. In contrast, studies on MS contributed the fewest foci and showed the second-lowest average study quality as well as the lowest average fatigue severity.

Additionally, Fig. 5D provides an overview of study design by disorder category. Most studies were task-based, with the exception of those on PD, where only resting-state studies were identified.

## Discussion

### Summary of systematic literature review

Cognitive or mental fatigue is a critical symptom affecting mental health, with significant implications for daily functioning and overall well-being. In this regard, it is a significant symptom across various diseases and disorders. Currently, it has gained considerable attention as a persistent and debilitating symptom following COVID-19 infections, affecting recovery and overall quality of life. Our systematic review corroborated the transdiagnostic importance of fatigue, revealing numerous studies investigating functional brain correlates in patients with a wide range of diagnoses.

In the reviewed studies eligible for the meta-analysis, the most prevalent clinical contexts were chronic fatigue syndrome/myalgic encephalomyelitis (CFS/ME), MS, and TBI. Other disorders, such as GWI, Post–COVID-19 condition (PC), PD, growth hormone deficiency (GHD), hepatitis C virus (HCV), breast cancer (BC), insomnia disorder (ID), fibromyalgia (FM) and Lyme disease, were scarcely represented. Surprisingly, only a few PC studies had been published at the time of our literature scan. This may be due to the fact that we excluded certain types of publications, such as case reports, preprints and opinions, and the major scientific research on this condition is likely still to be published.

Finally, a total of 47 neuroimaging studies were found eligible during the systematic literature review, all published within the past two decades (2004–2024), with the majority appearing in the last 10 years (*n* = 36). We screened 28 task-based functional brain designs as well as 28 resting state ones. The following meta-analysis synthesizes their results, involving a total of 2661 individuals. This substantial body of scientific output underscores the clinical relevance of mental fatigue and reflects the growing use of functional neuroimaging to explore its brain correlates.

### The mental fatigue network across disorders

Regardless of the clinical diagnoses focused in the neuroimaging studies, we found that mental fatigue is associated with a distributed cortical–subcortical network, including frontal and parietal areas, limbic structures (cingulate, thalamus and insular cortex), and ultimately basal ganglia areas (caudate and lentiform nuclei).

Different studies have suggested the role of cortical–striatal structures interplay during mental and cognitive fatigue.^96^ Specifically, failed non-motor functions of basal ganglia and their impact on the flow of information within the basal ganglia system and the striato-thalamo-cortical feedback loop, have been suggested to contribute to mental fatigue both in healthy and clinical populations.^96,97^ This mechanism arises from an inappropriate effort output and outcome evaluation due to cortico-striatal network (e.g. frontal and basal ganglia regions) deviating from normal functioning. While healthy subjects rely less and less on basal ganglia recruitment during mental fatigue tasks, in clinical samples (e.g. MS and TBI) the recruitment of striatal structures remains constant at the expense of frontal additional resources needed to complete a cognitive task.^96,98^ These findings outline how frontal compensatory mechanisms are needed in clinical conditions affected by mental fatigue symptoms, with basal ganglia as central structures in supporting cognitive fatigue.^96^ Together with these results, other studies suggested that fatigue might occur as a result of reduced DA availability in the striatal structures, thus leading to reduced firing of striatal DA neurons: indeed, basal ganglia structures are involved not only in effort–reward decision-making processes, but also to the feeling of fatigability during cognitive processes:^96,99,100^

Frontal, cingulate and parietal regions are overall demonstrated to be involved in the pathophysiology of chronic mental fatigue, specifically leading to attentional, executive and working memory (WM) deficits which characterize mental fatigue profiles.^60^ Specifically, posterior parietal regions have been demonstrated to play an important role during WM tasks for the short-term storage and retrieval of information, as well as shifting-executive functions.^60,101,102^ Mental fatigue symptoms might be related to cognitive deficits during the attending, store or shift processes of a stimulus, overall comprising broad attentional, mnestic and executive disturbances.^60,103^ Together with these results, praecuneus is an essential node of the DMN and it is involved in visual perception, episodic memory retrieval, self-processing and consciousness functions.^104^ Located in the medial posterior parietal lobe, it is thought to be critical for a number of neurocognitive functions. The over recruitment of this DMN hub has been demonstrated to be significant as a facilitation and compensatory factor during mental or cognitive demanding tasks.^105^ This DMN area has been proposed also as body fatigue driving factor, which play an important role for introspection and self-perceived level of fatigue, leading to an increase of inner thoughts regarding own perceived fatigue.^106,107^ Another important parietal region linked to cognitive fatigue is the angular gyrus. The angular gyrus activation is related to visuospatial and attentional mechanisms, sustaining the sense of agency.^108^ A common trend reported by studies investigating fatigue is the involvement of parietal cortices, especially the IPL (e.g. angular gyrus) in self-reported levels of fatigue.^61^ Due to the involvement of parietal regions in multisensory stimuli gating and encoding, as well as attentional processes, their involvement in cognitive tasks suggest a pronounced level of fatigue and the difficulty of being engaged in such tasks for clinical samples.^61^

Together with parietal regions, the cingulate cortex participates in specific memory and executive mechanisms, which lead to memory overloads and executive deficits as primary symptoms of mental fatigue.^60,101,109^ At the same time, cingulate structures are implicated in inward attention and mental states shifts that may lead to a greater attentional internal focus when experiencing chronic mental fatigue.^60,110^

As regards the experimental design influence in defying and forming each of the three fatigue network clusters, we found that the majority of the eligible studies are task-based, with only a small number of studies classified as resting state. Of note, the ratio of the number of tasks and resting state studies involved in shaping each cluster is similar. Thus, we can draw the conclusion that none of the clusters revealed by the meta-analysis was driven by one specific experimental study-design.

Overall, these results strengthen our investigation premise based on the fact that a set of brain regions forms a functional network responsible for maintaining fatigue. This network may be abnormal both at rest and while performing any kind of neuropsychological task. Thus, we believe that our study has the potential of outlining a functional network that can be linked to mental fatigue as a transdiagnostic symptom: according to studies retrieved, brain areas sustaining fatigue show patterns which are study-design and diagnosis independent.

### Diagnose-specific ALE results

In line with these general results, an investigation on specific disorders which are shown to be prominently present in our literature screening (GWI, MS, PD, TBI, CFS) resulted in a similar pattern of findings. Each of these disorders share commonalities in the activations of most of the main cortical–subcortical fatigue-related hubs, that can be associated to the three above-mentioned clusters, such as frontal, basal ganglia, limbic, parietal and temporal structures. Therefore, the clusters appear to consistently represent brain areas related with mental fatigue across different diagnoses. Notably, in all five diagnosis-specific groups, the frontal–striatal–limbic and frontal–cingulate clusters were prominent. Although their proportions varied, these two clusters together comprised the majority of the foci included in the meta-analysis. The cingulate cortex, along with premotor and frontal areas, forms a cluster more related to executive and memory mechanisms, which are also altered in mental fatigue.^60,107,110,111^ Overall, cingulate structures play an important role in the self-evaluation of mental fatigue symptoms, acting as a central cortical hub for interoceptive self-awareness of fatigue across multiple neural systems.^111,112^ Overall, the frontal–striatal–limbic cluster seemed to be especially relevant in the context of mental fatigue, as the number of foci contributed in our meta-analysis was comparably large and homogeneous across studies and diagnoses. The role of the cortical and subcortical structures found in this cluster can be interpreted in light of the dual *regulation system* model of mental fatigue.^111^ This model posits that mental fatigue can be described by the interplay of two parallel mechanisms, evident during cognitive or mental function tasks: facilitation and inhibition.^111^ Specifically, a thalamic–frontal loop—which interconnects striatal, limbic system, frontal and thalamic areas—supports a mental facilitation system needed to maintain a cognitive performance despite mental fatigue symptoms; on the other hand, mental overload activates the so-called inhibition system. This latter process is sustained by the involvement of insular and cingulate cortex regions, which resulted in evident cognitive performance impairments at the expense of the motivational and facilitation mechanisms ruled by a thalamic–striatal–frontal loop.^111^ The shift from an acute to chronic mental fatigue condition is thus explained by an overactivation of the inhibition–limbic system during time, with less cognitive (attentional, memory and executive functions) facilitation resources available.^111^ The parietal counterpart of the attentional system is involved in attentional modulation and inhibition functions, which seem to be disrupted in mental fatigue, leading to a decline in cognitive performance and a feeling of exhaustion.^113,114^ The interaction of parietal and frontal areas seems to change over time facilitating chronic mental fatigue.^115^ While top-down attentional mechanisms from frontal to parietal regions remain intact, chronic fatigue increases parietal influence on frontal areas, suggesting a shift toward somatosensory and emotional inward attention and greater network segregation.^115^ Altered connectivity with brain regions associated with motor function, cognitive control and sensory processing, such as the supplementary motor area, frontal regions and thalamus, is significant in mental fatigue disorders like post-viral diseases.^81^

Although we observed significant commonalities in the prominent expression of the three-key fatigue-related clusters across disease categories, notable differences emerge depending on the disorder’s aetiology, supporting a differential involvement of each neural network. A pivotal role of the frontal–striatal–limbic cluster was evident across MS, TBI, and PD, though with disease-specific patterns and additional contributions from other networks.

A key feature common to all three disorders is impaired striatal connectivity, which is essential for reward processing and goal-directed behaviour.^55,87^ These findings align with the dopamine imbalance hypothesis of fatigue.^53,55,63,116^ Dysfunctions in limbic areas, including the insula and anterior cingulate cortex, have been proposed to contribute to both fatigue and related emotional symptoms, such as depression.^85,86,88^

Overall, dysfunction within the thalamo–cortical–striatal loop, modulated by both DA and serotonergic systems, may underlie the persistent emotional and motivational deficits observed in fatigue, particularly through disrupted frontal–striatal communication.^97,116^ Despite their distinct pathophysiologies, all three disorders share common structural brain alterations, supporting the plausibility of a shared aetiology for fatigue. Specifically, disruptions in brain regions involved in emotional regulation, motivation, and cognitive control are observed across conditions. Altered connectivity in the thalamic, striatal and frontal regions reflects impaired top-down control and effort regulation.

In contrast to conditions like MS, TBI, and PD, where altered brain structures are central to the ethology of fatigue, CFS and GWI do not primarily involve structural brain changes. Instead, these disorders are characterized by persistent, unexplained fatigue as a hallmark symptom. In CFS and GWI, the frontal–cingulate cluster plays a more prominent role in fatigue-related dysfunctions. Of note, the cingulate cortex is a critical node in the central executive network, implicated in cognitive processes such as attention, inhibition, and the maintenance of information.^61^ In patients with CFS and CFS, alterations in this network seem to be related to impaired top-down cognitive control and motivation.^70,75-77^

The parietal cluster appears to play a particularly prominent role in (GWI). Parietal regions are crucial for integrating multisensory information, coordinating movement, and allocating attentional resources. Besides cognitive fatigue, dysfunction in these areas may underlie several core symptoms of GWI, including attentional deficits, motor control impairments and pain perception.^70,75,76^ Notably, the parietal cluster was not implicated in the PD group, which may be attributed to the exclusively resting-state design of the included PD studies—unlike the predominantly task-based designs used in the other five diagnostic groups. This methodological difference may underscore the parietal cortex's role in attention allocation during active task performance.^94^

### Potential influencing factors

Finally, we considered several potential factors that may have influenced our overall findings. One key factor is the methodological quality of the included studies. Overall, the majority of studies demonstrated a moderate risk of bias, as indicated by total scores ranging between 4 and 6 on the NOS. Specifically, considering the five diagnostic groups we analysed more closely (CFS, PD, TBI, MS and GWI), the quality ratings were quite similar. Nevertheless, the comparison between each of the diagnostic categories highlights also that, on average, the group of studies on TBI has the highest quality score, indicating the lowest risk of bias. These studies also contributed the largest number of foci to the overall results. In contrast, studies on MS contributed the fewest foci to the mental fatigue network definition and showed the second-lowest average study quality.

Considering fatigue severity as another potential influencing factor on our results, we estimated effect sizes based on comparisons of fatigue levels between clinical samples and HCs as reported in the studies. These effect sizes varied considerably, both across and within diagnostic groups. When examining the average Cohen’s *d* values, MS patients exhibited the lowest levels of fatigue severity. This may be attributable to the fact that the MS group contributed the fewest activation foci in the analysis. In contrast, the effect sizes indicated that PD patients had the highest fatigue severity. Interestingly, CFS patients reported relatively low fatigue severity, which was surprising given that fatigue is a hallmark symptom of CFS. This discrepancy may be attributed to the heterogeneity in the quantitative assessments used and the factorial design of their scales. Specifically, while all PD studies used the same fatigue measure, the Fatigue Severity Scale, none of the CFS studies utilized this tool. Instead, most of the CFS studies relied on the Chronic Fatigue Scale (ChFS) as a measure of mental fatigue. Given that the ChFS has been reported to be susceptible to ceiling effects, it is possible that the sensitivity and specificity of the quantitative fatigue assessments played a critical role in these findings.^117^

In summary, the heterogeneity in study design, methodological quality and the fatigue severity of the patient samples posed limitations that may have obscured a clear interpretation of their influence on our results.

### Limitations

This study has several limitations that we wish to acknowledge. First, we were unable to control for the overrepresentation of certain diseases and study designs compared to others within the study pool. Second, some disorders (e.g. TBI, PD) do not primarily define mental fatigue as a central factor, unlike other conditions such as CFS. Therefore, comorbid pathological endophenotypes should be considered. Third, while we focused on functional alterations related to mental fatigue in patients compared to HCs, we did not assess whether these alterations represented statistically significant increases or decreases. The ALE-analysis also combined different methodologies used for acquiring (PET and fMRI) and analysing functional brain data (e.g. functional connectivity or task activations) across studies. This hinders a consistent interpretation on directionality. Instead, the ALE analysis focuses on the location of regions that have been reported to be linked to mental fatigue in order to assess brain regions most likely to be involved in the development and maintenance of mental fatigue. Fourth, the majority of the studies retrieved and classified as eligible are task-based in their experimental design. Finally, we acknowledge two modifications to our pre-registered procedures. First, as noted, we revised the eligibility criterion for fatigue assessment to exclude behavioural measures. Second, we applied family-wise error correction at the cluster level rather than the voxel level, as recommended by Eickhoff and colleagues.^50^

## Conclusion

Our analysis highlights the pivotal roles of three distinct spatial clusters in mental fatigue: the frontal–striatal–limbic, frontal–cingulate, and parietal clusters, each representing different facets of the fatigue experience.

Altered activation patterns in the parietal and frontal–cingulate regions point to an increased focus on somatosensory and interoceptive signals, suggesting that individuals with mental fatigue may become hyper-attuned to their own visceral sensations. This shift likely reflects a maladaptive mechanism tied to the inhibition system, heightening inward attention and emotional reactivity. In addition, disruptions in the thalamic-striatal-frontal loop, linked to the facilitation system, may further exacerbate fatigue symptoms by contributing to a decline in cognitive and emotional resources. Collectively, these findings offer a comprehensive view of the brain regions involved in mental fatigue across various disorders.

## Supplementary Material

fcaf315_Supplementary_Data

## Data Availability

The preregistration can be found at the International prospective register of systematic reviews PROSPERO at https://www.crd.york.ac.uk/prospero/display_record.php?ID= CRD42023414657. For additional review materials, please contact the corresponding author.
