# Correction to: Mapping the brain’s fatigue network: a transdiagnostic systematic review and meta-analysis on functional correlates of mental fatigue

**DOI:** 10.1093/braincomms/fcag194

**Published:** 2026-06-03

This is a correction to: Andy Schumann, Monica Di Giuliano, Steffen Schulz, Feliberto de la Cruz, Teresa Kreuder, Georg Seifert, Karl-Jürgen Bär, Mapping the brain’s fatigue network: a transdiagnostic systematic review and meta-analysis on functional correlates of mental fatigue, *Brain Communications*, Volume 7, Issue 5, 2025, fcaf315, https://doi.org/10.1093/braincomms/fcaf315

In the originally published version of this manuscript, the caption of Figure 4 contained labels that were ordered incorrectly.

These have now been corrected to read:

“**A** traumatic brain injury (TBI, 6 studies, 343 subjects, 168 foci), **B** Parkinson’s disease (PD, 3 studies, 210 subjects, 38 foci), **C** multiple sclerosis (MS, 12 studies, 585 subjects, 178 foci), **D** Gulf War illness (GWI, 3 studies, 270 subjects, 116 foci), **E** chronic fatigue syndrome (CFS, 14 studies, 630 subjects, 217 foci)”.

Instead of:

“**A** Gulf War illness (GWI, 3 studies, 270 subjects, 116 foci), **B** multiple sclerosis (MS, 12 studies, 585 subjects, 178 foci), **C** Parkinson’s disease (PD, 3 studies, 210 subjects, 38 foci), **D** traumatic brain injury (TBI, 6 studies, 343 subjects, 168 foci), **E** chronic fatigue syndrome (CFS, 14 studies, 630 subjects, 217 foci)”.

