# Supplementary material for: Mapping the brain’s fatigue network: a transdiagnostic systematic review and meta-analysis on functional correlates of mental fatigue
Source: Brain Commun. 2025 Aug 28;7(5):fcaf315. doi: 10.1093/braincomms/fcaf315 (PMC12421237; doi:10.1093/braincomms/fcaf315)
Supplement: fcaf315_Supplementary_Data [file fcaf315_supplementary_data.docx]

**Mapping the brain’s fatigue network: a transdiagnostic systematic review and meta-analysis on functional correlates of mental fatigue**

Andy Schumann, Monica Di Giuliano, Steffen Schulz, Feliberto de la Cruz, Teresa Kreuder, Georg Seifert, Karl- Jürgen Bär

The pooled analysis of studies which focused on mental fatigue networks across all the disorders with a minimal number of 3 studies per category showed a widespread cortical-subcortical map, with some commonalities and differences in the functional activations across all the diseases **(Table S1)**.

**Table S1** ALE clusters according to different clinical categories.

| **Anatomical region** | **Size (mm^3^)** | **L/R** | **Max ALE score** | **MNI coordinates** |
| --- | --- | --- | --- | --- |
| **Gulf war illness (4 studies, 412 subjects, 117 foci, 8.76 mm FWHM)** | | | | |
| Medial frontal gyrus | 3320 | R/L | 0.0119/0.009 | 6 -2 64/-4 4 62 |
| Superior frontal gyrus | 2136 | R | 0.0106 | 22 30 44 |
| Middle frontal gyrus | 1744 | R/L | 0.0119/0.0094 | 52 32 14/-33 33 35 |
| Inferior frontal gyrus | 1088 | R/L | 0.0097/0.0091 | 50 15 21/ -48 12 18 |
| Postcentral gyrus | 1088 | R/L | 0.0094/0.0097 | 41 -25 53/-42 -22 48 |
| Precentral gyrus | 1088 | L | 0.0097 | -38 -6 50 |
| Middle temporal gyrus | 1088 | R | 0.0094 | 57 -37 -1 |
| Transverse temporal gyrus | 1088 | L | 0.0094 | -52 -20 8 |
| Lingual gyrus | 1088 | R/L | 0.0097/0.0097 | 16 -73 9/-7 -79 6 |
| Cuneus | 1992 | R | 0.0101 | 14 -79 28 |
| Middle occipital gyrus | 1088 | R | 0.0097 | 37 -80 19 |
| Insula | 3176 | R/L | 0.0097/0.0146 | 53 -6 15/-44 -6 12 |
| Lentiform nucleus | 2992 | L | 0.0098 | -16 14 -4 |
| Caudate nucleus | 2992 | R/L | 0.0091/0.0098 | 15 12 9/-14 20 -8 |
| Thalamus | 2216 | L | 0.0108 | -12 -18 8 |
| Cingulate gyrus | 2168 | R/L | 0.010/0.0098 | 14 30 44/-12 24 40 |
| Anterior cingulate gyrus | 1968 | R/L | 0.0105/0.0026 | 18 48 -14/2 36 15 |
| Parahippocampal gyrus | 1088 | L | 0.0097 | -22 -16 -22 |
| Anterior Lobe | 2176 | R | 0.0105 | 24 -46 -32 |
| Culmen - Anterior Lobe | 2176 | L | 0.0105 | 2 -52 -6 |
| Nodule - Anterior Lobe | 1088 | R | 0.0097 | 2 -55 -35 |
| Posterior Lobe | 2008 | L | 0.0062 | -28 -57.5 -46.5 |
| Tonsil - Posterior Lobe | 2008 | R | 0.0101 | 26 -56 -48 |
| Tuber - Posterior Lobe | 1088 | L | 0.0094 | -35 -67 -29 |
| **Multiple sclerosis (12 studies, 585 subjects, 178 foci, 12.9 mm FWHM)** | | | | |
| Medial frontal gyrus | 1336 | L | 0.0099 | -8 52 6 |
| Middle frontal gyrus | 4520 | R/L | 0.0094/0.0126 | 48 8 42/-30 4 46 |
| Inferior frontal gyrus | 2576 | L | 0.0105 | -44 22 4 |
| Precentral gyrus | 4520 | R/L | 0.0132/0.0117 | 44 4 28/-28 -12 72 |
| Postcentral gyrus | 3248 | L | 0.0149 | -58 -26 20 |
| Superior parietal lobule | 1552 | L | 0.0092 | -32 -52 54 |
| Paracentral gyrus | 1288 | L | 0.0123 | 2 -22 52 |
| Precuneus | 1320 | L | 0.0101 | -18 -42 64 |
| Fusiform | 3824 | L | 0.0103 | -36 -54 -10 |
| Superior temporal gyrus | 1600 | R | 0.0092 | 56 -28 12 |
| Middle temporal gyrus | 1088 | L | 0.0098 | -60 -26 -12 |
| Inferior temporal gyrus | 1220 | R | 0.0097 | 59.3 -16 -29.3 |
| Parahippocampal gyrus | 3824 | L | 0.0101 | -26 -48 -12 |
| Cingulate gyrus | 1592 | R/L | 0.0096/0.0138 | 14 28 32/-2 10 26 |
| Anterior cingulate gyrus | 1336 | R | 0.0093 | 6 28 22 |
| Posterior cingulate gyrus | 2344 | R | 0.0177 | 6 -42 16 |
| Caudate nucleus | 4280 | R/L | 0.0134/0.0120 | 20 -8 22/ -16 14 8 |
| Lentiform nucleus | 4280 | R/L | 0.0104/0.0103 | 16 2 4/-28 0 -12 |
| Claustrum | 2680 | L | 0.0105 | -36 8 -14 |
| Thalamus | 4280 | R | 0.0106 | 14 -6 10 |
| Insula | 3284 | R/L | 0.0119/0.0136 | 36 12 10/-46 -20 16 |
| Culmen - Anterior Lobe | 3824 | L | 0.0102 | -16 -54 -10 |
| Declive - Posterior Lobe | 3824 | R/L | 0.0092/0.0102 | 22 -70 -24/-16 -58 -12 |
| Inferior semilunar lobule - Posterior Lobe | 2488 | R/L | 0.0185/0.0104 | 40 -70 -50/-26 -82 -36 |
| Tuber - Posterior Lobe | 2488 | L | 0.0104 | -36 -70 -30 |
| Pyramis - Posterior Lobe | 2488 | R/L | 0.104/0.0092 | 32 -72 -32/-22 -74 -28 |
| **Parkinson’s disease (3 studies, 210 subjects, 38 foci, 8.69 FWHM)** | | | | |
| Medial frontal gyrus | 8360 | L | 0.0095 | -24 40 16 |
| Superior frontal gyrus | 11232 | R/L | 0.0099/0.0094 | 30 48 10/-38 46 24 |
| Middle frontal gyrus | 8360 | L | 0.0095 | -30 50 16 |
| Inferior frontal gyrus | 11232 | R | 0.0095 | 42 38 4 |
| Inferior parietal lobule | 5264 | L | 0.0099 | -36 -63 48 |
| Precuneus | 5264 | L | 0.0084 | -39 -74 44 |
| Precentral gyrus | 5864 | R/L | 0.0095/0.0084 | 22 -21 75/-54 -4.5 19.5 |
| Postcentral gyrus | 2832 | R | 0.0099 | 27 -24 72 |
| Superior temporal gyrus | 2952 | L | 0.0097 | -58 -42 18 |
| Transverse gyrus | 2848 | R | 0.0095 | 57 -23 12 |
| Sub-gyral temporal area | 2944 | R | 0.0099 | 45 -12 -12 |
| Superior occipital gyrus | 5264 | L | 0.0102 | -40 -76 36 |
| Cingulate gyrus | 8112 | R/L | 0.0091/0.0093 | 13 -33 45/-8 6 51 |
| Anterior cingulate gyrus | 2920 | L | 0.0102 | 0 48 0 |
| Posterior cingulate gyrus | 2944 | R | 0.0097 | 3 -48 18 |
| Insula | 11232 | R/L | 0.0095/0.0099 | 36 28 8/-38 18 1 |
| Tonsil - Posterior Lobe | 2976 | L | 0.0093 | -30 -45 -51 |
| **Traumatic brain injury (6 studies, 343 subjects, 168 foci, 9.24 mm FWHM)** | | | | |
| Medial frontal gyrus | 2152 | R | 0.0135 | 4 6 56 |
| Superior frontal gyrus | 2152 | R/L | 0.0104/0.0123 | 4 24 48/-20 56 34 |
| Middle frontal gyrus | 1584 | R/L | 0.0181/0.0104 | 50 38 16/-36 8 52 |
| Inferior frontal gyrus | 3912 | R/L | 0.0100/0.0133 | 54 30 12/-46 26 10 |
| Precentral gyrus | 1016 | L | 0.0097 | -54 2 34 |
| Postcentral gyrus | 1064 | L | 0.0110 | -50 -18 56 |
| Superior parietal lobule | 3544 | R | 0.0110 | 32 -68 50 |
| Inferior parietal lobule | 2680 | L | 0.0126 | -44 -42 48 |
| Precuneus | 1400 | R/L | 0.0126/0.0104 | 38 -72 46/-38 -68 40 |
| Parietal sub-gyral area | 3544 | R | 0.0096 | 36 -60 40 |
| Angular gyrus | 3408 | L | 0.0094 | -30 -56 42 |
| Supramarginal gyrus | 2680 | L | 0.0098 | -44 -36 40 |
| Fusiform gyrus | 3576 | L | 0.0154 | -40 -52 -18 |
| Superior temporal gyrus | 3408 | L | 0.0099 | -40 -52 36 |
| Middle temporal gyrus | 1152 | L | 0.0105 | -38 -62 34 |
| Cuneus | 3352 | L | 0.0144 | -6 -72 10 |
| Lingual gyrus | 3352 | L | 0.0098 | -10 -60 4 |
| Inferior occipital gyrus | 1024 | L | 0.0097 | -16 -94 -4 |
| Thalamus | 5616 | R/L | 0.0135/0.0206 | 8 -18 10/-12 -12 2 |
| Caudate nucleus | 5616 | R/L | 0.0106/0.0136 | 10 16 6/-12 12 6 |
| Lentiform nucleus | 2096 | R | 0.0195 | 18 12 0 |
| Insula | 3912 | L | 0.0113 | -34 24 0 |
| Claustrum | 3912 | L | 0.0113 | -32 18 6 |
| Parahippocampal gyrus | 3352 | L | 0.0104 | -20 -54 0 |
| Anterior cingulate gyrus | 2632 | R/L | 0.0091/0.0104 | 4 42 20/-6 34 24 |
| Posterior cingulate gyrus | 1400 | R/L | 0.0106/0.0104 | 20 -54 20/-6 -52 20 |
| Cingulate gyrus | 1344 | R/L | 0.0098/0.0107 | 8 28 38/-4 0 50 |
| Anterior Lobe | 3576 | L | 0.0102 | -30 -54 -32 |
| Culmen - Anterior Lobe | 3576 | L | 0.0116 | -38 -52 -28 |
| **Chronic fatigue syndrome/myalgic encephalitis (13 studies, 630 subjects, 216 foci, 9.28 mm FWHM)** | | | | |
| Precentral gyrus | 2432 | L | 0.0141 | -36 -2 52 |
| Postcentral gyrus | 1288 | R | 0.0105 | 44 -26 36 |
| Medial frontal gyrus | 1968 | R | 0.0090 | 12 20 40 |
| Superior frontal gyrus | 1968 | R/L | 0.0089/0.0090 | 44 48 22/-4 18 48 |
| Middle frontal gyrus | 2432 | R/L | 0.0098/0.0097 | 40 44 16/-44 4 50 |
| Inferior frontal gyrus | 1544 | R/L | 0.0148/0.0090 | 48 22 4/-44 8 34 |
| Superior temporal gyrus | 1120 | R/L | 0.0157/0.0113 | 58 -28 8/-52 -6 -4 |
| Middle temporal gyrus | 5368 | R/L | 0.0108/0.0091 | 34 -66 30/-48 -66 4 |
| Inferior temporal gyrus | 5368 | L | 0.0086 | -50 -60 -2 |
| Temporal sub-gyral | 1336 | R | 0.0098 | 38 -36 -10 |
| Fusiform gyrus | 5368 | L | 0.0138 | -50 -58 -14 |
| Superior parietal lobule | 3744 | R | 0.0090 | 34 -54 46 |
| Inferior parietal lobule | 1288 | R | 0.0097 | 40 -32 32 |
| Angular gyrus | 6160 | R | 0.0090 | 32 -54 42 |
| Precuneus | 6160 | R/L | 0.0090/0.0177 | 32 -68 34/-26 -66 38 |
| Cingulate gyrus | 2608 | R/L | 0.0096/0.0113 | 4 14 40/-10 8 40 |
| Posterior cingulate gyrus | 2072 | R/L | 0.0097/0.0123 | 24 -64 18/-6 -30 26 |
| Lingual gyrus | 2688 | R/L | 0.0110/0.0107 | 18 -76 10/-20 -78 4 |
| Cuneus | 2072 | R | 0.0090 | 22 -74 20 |
| Middle occipital gyrus | 5368 | R | 0.0112 | 24 -92 -2 |
| Inferior occipital gyrus | 1784 | R | 0.0100 | 32 -94 0 |
| Thalamus | 1968 | L | 0.0101 | -14 -22 8 |
| Insula | 3424 | R | 0.0129 | 60 -30 20 |
| Claustrum | 1368 | R | 0.0098 | 38 -8 -4 |
| Parahippocampal gyrus | 1336 | R | 0.0113 | 30 -40 -8 |
| Lentiform nucleus | 1368 | R | 0.0099 | 30 -10 -8 |
| Caudate | 1816 | L | 0.0096 | -16 -10 24 |
| Nodule - Anterior Lobe | 1664 | R | 0.0086 | 10 -68 -28 |
| Culmen - Anterior Lobe | 1568 | R/L | 0.103/0.0119 | 12 -36 12/0 -62 4 |
| Declive - Posterior Lobe | 5368 | L | 0.0094 | -46 -58 -22 |
| Pyramis - Posterior Lobe | 1664 | R | 0.0093 | 8 -76 -26 |

p < 0.05 is the chosen p value threshold; Min. Volume (mm**^3^**) of 1000 is the cluster threshold for forming the minimum cluster size; uncorrected for FWE. L, Left; R, right.

We used the Newcastle-Ottawa Scale (NOS) to assess the risk of bias in the studies that we have analyzed here. A NOS score ranging from 7 to 9 indicates a study with high quality and a low risk of bias, while studies with a score below four have the highest risk of bias. Most of the studies we have analyzed in this work had a moderate risk of bias (NOS 4-6) with a maximum score of eight and minimum score of three. While the number of response options varies across items, only the highest-quality responses receive a star, with no points awarded for lower levels. Importantly, none of the studies scored on the item *ascertainment of exposure*, as this requires external assessment via medical records or a structured interview. Since fatigue is challenging to evaluate by another person, individuals rated their fatigue levels subjectively. We found that behavioral proxies of cognitive fatigue, such as time-on-task or reaction time, are not well standardized or generally accepted. This is why we decided (in contradiction to the pre-registration) that the assessment of fatigue should be performed via scales such as VAS or questionnaires. Other frequent reasons for lower NOS scores included a brief and, therefore, insufficient description of control selection and the independent validation of diagnoses, which might have been incorporated more often than explicitly stated in the articles.

**Table S2** Risk of bias assessment according to the Newcastle-Ottawa scale. All nine items of the Newcastle-Ottawa scale rated with either a star (★) or zero points (-). The total score is the sum of all stars. Item S1: Definition of cases; Item S2: Representativeness of cases; Item S3: Selection of controls; Item S4: Definition of controls; Item E1: Ascertainment of exposure; Item E2: Same method for cases and controls; Item E3: Non-response rate; Item C1a: Controlled for most important factor; Item C1b: Controlled for additional factor(s). a: potential for selection bias of cases due to a set minimum or maximum symptom severity (other than fatigue) b: potential for selection bias of cases due to set minimum fatigue severity.

| **Authors** | **Selection** | | | | **Exposure** | | | **Comparability** | | **Total**  **Score** |
| --- | --- | --- | --- | --- | --- | --- | --- | --- | --- | --- |
|  | ***S1*** | ***S2*** | ***S3*** | ***S4*** | ***E1*** | ***E2*** | ***E3*** | ***C1a*** | ***C1b*** |  |
| Alshelh et al., 2020 | **-** | **★** | **★** | **★** | **-** | **★** | **★** | **-** | **-** | **5** |
| Amann et al., 2011 | **★** | **-***a* | **-** | **★** | **-** | **★** | **★** | **-** | **-** | **4** |
| Arwert et al., 2005 | **-** | **★** | **★** | **-** | **-** | **★** | **★** | **-** | **-** | **4** |
| Berginström et al., 2018 | **-** | **-** | **★** | **★** | **-** | **★** | **★** | **★** | **-** | **5** |
| Boissoneault et al., 2016 | **-** | **★** | **★** | **-** | **-** | **★** | **★** | **-** | **-** | **4** |
| Boissoneault et al., 2018 | **-** | **★** | **★** | **-** | **-** | **★** | **★** | **-** | **-** | **4** |
| Bruijel et al., 2022 | **★** | **★** | **★** | **-** | **-** | **★** | **★** | **★** | **★** | **7** |
| Cagna et al., 2023 | **-** | **-***b* | **★** | **-** | **-** | **★** | **★** | **★** | **-** | **4** |
| Caseras et al., 2006 | **★** | **★** | **★** | **★** | **-** | **★** | **★** | **-** | **-** | **6** |
| Caseras et al., 2008 | **★** | **★** | **★** | **★** | **-** | **★** | **★** | **-** | **-** | **6** |
| Chen et al., 2020 | **-** | **★** | **★** | **-** | **-** | **★** | **★** | **-** | **-** | **4** |
| Cook et al., 2007 | **★** | **★** | **★** | **-** | **-** | **★** | **★** | **-** | **-** | **5** |
| Cook et al., 2017 | **★** | **★** | **-** | **-** | **-** | **★** | **★** | **-** | **-** | **4** |
| de Lange et al., 2004 | **-** | **★** | **★** | **-** | **-** | **★** | **★** | **-** | **-** | **4** |
| Dobryakova et al., 2020 | **★** | **-***b* | **★** | **★** | **-** | **★** | **★** | **-** | **-** | **5** |
| Fallon et al., 2009 | **-** | **★** | **-** | **-** | **-** | **★** | **★** | **★** | **★** | **5** |
| Gay et al., 2016 | **-** | **★** | **-** | **★** | **-** | **★** | **★** | **-** | **-** | **4** |
| Genova et al., 2013 | **-** | **★** | **-** | **-** | **-** | **★** | **★** | **-** | **-** | **3** |
| Glass et al., 2011 | **★** | **★** | **-** | **-** | **-** | **★** | **★** | **★** | **-** | **5** |
| Guo et al., 2023 | **-** | **★** | **★** | **★** | **-** | **★** | **★** | **★** | **★** | **7** |
| Heeren et al., 2011 | **-** | **★** | **-** | **-** | **-** | **★** | **★** | **-** | **-** | **3** |
| Hesse et al., 2014 | **-** | **★** | **★** | **-** | **-** | **★** | **★** | **-** | **-** | **4** |
| Li et al., 2017 | **-** | **-***a* | **★** | **-** | **-** | **★** | **★** | **★** | **★** | **5** |
| Lin et al., 2019 | **-** | **★** | **★** | **-** | **-** | **★** | **★** | **★** | **★** | **6** |
| Liu et al., 2016 | **-** | **★** | **-** | **-** | **-** | **★** | **★** | **★** | **-** | **4** |
| Menning et al., 2017 | **-** | **★** | **★** | **-** | **-** | **★** | **★** | **-** | **-** | **4** |
| Mizuno et al., 2015 | **★** | **★** | **★** | **-** | **-** | **★** | **★** | **-** | **-** | **5** |
| Nordin et al., 2016 | **-** | **-***b* | **-** | **-** | **-** | **★** | **★** | **★** | **★** | **4** |
| Provenzano et al., 2020 | **-** | **★** | **-** | **-** | **-** | **★** | **★** | **★** | **★** | **5** |
| Shan et al., 2023 | **-** | **-***a* | **★** | **-** | **-** | **★** | **★** | **★** | **★** | **5** |
| Spiteri et al., 2019 | **-** | **★** | **-** | **-** | **-** | **★** | **★** | **-** | **-** | **3** |
| Staud et al., 2018 | **-** | **★** | **★** | **★** | **-** | **★** | **★** | **-** | **-** | **5** |
| Svolgaard et al., 2018 | **-** | **-a** | **★** | **-** | **-** | **★** | **★** | **★** | **★** | **5** |
| Svolgaard et al., 2022 | **-** | **★** | **-** | **-** | **-** | **★** | **★** | **★** | **★** | **5** |
| Wagner et al., 2022 | **-** | **-***a* | **★** | **★** | **-** | **★** | **★** | **-** | **-** | **4** |
| Wang et al., 2021 | **-** | **★** | **★** | **-** | **-** | **★** | **★** | **★** | **★** | **6** |
| Washington et al., 2020a | **-** | **★** | **-** | **-** | **-** | **★** | **★** | **-** | **-** | **3** |
| Washington et al., 2020b | **-** | **★** | **-** | **-** | **-** | **★** | **★** | **★** | **★** | **5** |
| Wortinger et al., 2016 | **★** | **★** | **★** | **★** | **-** | **★** | **★** | **-** | **-** | **6** |
| Wortinger et al., 2017a | **★** | **★** | **★** | **★** | **-** | **★** | **★** | **-** | **-** | **6** |
| Wortinger et al., 2017b | **★** | **★** | **★** | **★** | **-** | **★** | **★** | **★** | **★** | **8** |
| Wu et al., 2016 | **-** | **-***a* | **★** | **-** | **-** | **★** | **★** | **★** | **★** | **5** |
| Zhang et al., 2017 | **-** | **-***a* | **-** | **-** | **-** | **★** | **★** | **★** | **★** | **4** |
| Zhao et al., 2024 | **-** | **★** | **★** | **-** | **-** | **★** | **★** | **★** | **★** | **6** |
| Zhou et al., 2016 | **-** | **★** | **★** | **★** | **-** | **★** | **★** | **★** | **★** | **7** |
| Zunini et al., 2013 | **-** | **★** | **★** | **★** | **-** | **★** | **★** | **-** | **-** | **5** |

**References**

Alshelh, Z. et al. (2020) ‘In-vivo imaging of neuroinflammation in veterans with Gulf War illness.’, Brain, Behavior, and Immunity, 87, pp. 498–507. Available at: https://doi.org/10.1016/j.bbi.2020.01.020.

Amann, M. et al. (2011) ‘Altered functional adaptation to attention and working memory tasks with increasing complexity in relapsing-remitting multiple sclerosis patients.’, Human Brain Mapping, 32(10), pp. 1704–1719. Available at: https://doi.org/10.1002/hbm.21142.

Arwert, L.I. et al. (2006) ‘Effects of Growth Hormone Substitution Therapy on Cognitive Functioning in Growth Hormone Deficient Patients: A Functional MRI Study’, Neuroendocrinology, 83(1), pp. 12–19. Available at: https://doi.org/10.1159/000093337.

Berginström, N. et al. (2018) ‘Using functional magnetic resonance imaging to detect chronic fatigue in patients with previous traumatic brain injury: Changes linked to altered striato-thalamic-cortical functioning’, The Journal of Head Trauma Rehabilitation, 33(4), pp. 266–274. Available at: https://doi.org/10.1097/htr.0000000000000340

Boissoneault, J. et al. (2016) ‘Abnormal resting state functional connectivity in patients with chronic fatigue syndrome: an arterial spin-labeling fMRI study.’, Magnetic resonance imaging, 34(4), pp. 603–608. Available at: https://doi.org/10.1016/j.mri.2015.12.008.

Boissoneault, J. et al. (2018) ‘Static and dynamic functional connectivity in patients with chronic fatigue syndrome: use of arterial spin labelling fMRI.’, Clinical physiology and functional imaging, 38(1), pp. 128–137. Available at: https://doi.org/10.1111/cpf.12393.

Bruijel, J. et al. (2022) ‘Task-induced subjective fatigue and resting-state striatal connectivity following traumatic brain injury.’, NeuroImage. Clinical, 33, p. 102936. Available at: https://doi.org/10.1016/j.nicl.2022.102936.

Cagna, C.J. et al. (2023) ‘Altered functional connectivity during performance feedback processing in multiple sclerosis.’, NeuroImage. Clinical, 37, p. 103287. Available at: https://doi.org/10.1016/j.nicl.2022.103287.

Caseras, X. et al. (2006) ‘Probing the working memory system in chronic fatigue syndrome: a functional magnetic resonance imaging study using the n-back task.’, Psychosomatic medicine, 68(6), pp. 947–955. Available at: https://doi.org/10.1097/01.psy.0000242770.50979.5f.

Caseras, X. et al. (2008) ‘The neural correlates of fatigue: an exploratory imaginal fatigue provocation study in chronic fatigue syndrome.’, Psychological medicine, 38(7), pp. 941–951. Available at: https://doi.org/10.1017/S0033291708003450.

Chen, M.H. et al. (2020) ‘Neural mechanisms underlying state mental fatigue in multiple sclerosis: a pilot study.’, Journal of neurology, 267(8), pp. 2372–2382. Available at: https://doi.org/10.1007/s00415-020-09853-w.

Cook, D.B. et al. (2007) ‘Functional neuroimaging correlates of mental fatigue induced by cognition among chronic fatigue syndrome patients and controls.’, NeuroImage, 36(1), pp. 108–122. Available at: https://doi.org/10.1016/j.neuroimage.2007.02.033.

Cook, D.B. et al. (2017) ‘Neural consequences of post-exertion malaise in Myalgic Encephalomyelitis/Chronic Fatigue Syndrome.’, Brain, Behavior, and Immunity, 62, pp. 87–99. Available at: https://doi.org/10.1016/j.bbi.2017.02.009.

Dobryakova, E. et al. (2020) ‘Reward presentation reduces on-task fatigue in traumatic brain injury’, Cortex, 126, pp. 16–25. Available at: https://doi.org/10.1016/j.cortex.2020.01.003.

Fallon, B.A. et al. (2009) ‘Regional cerebral blood flow and metabolic rate in persistent lyme encephalopathy.’, Archives of General Psychiatry, 66(5), pp. 554–563. Available at: https://doi.org/10.1001/archgenpsychiatry.2009.29.

Gay, C.W. et al. (2016) ‘Abnormal Resting-State Functional Connectivity in Patients with Chronic Fatigue Syndrome: Results of Seed and Data-Driven Analyses’, Brain Connectivity, 6(1), pp. 48–56. Available at: https://doi.org/10.1089/brain.2015.0366.

Genova, H.M. et al. (2013) ‘Examination of cognitive fatigue in multiple sclerosis using functional magnetic resonance imaging and diffusion tensor imaging.’, PloS one, 8(11), p. e78811. Available at: https://doi.org/10.1371/journal.pone.0078811.

Glass, J.M. et al. (2011) ‘Executive function in chronic pain patients and healthy controls: Different cortical activation during response inhibition in fibromyalgia.’, The Journal of Pain, 12(12), pp. 1219–1229. Available at: https://doi.org/10.1016/j.jpain.2011.06.007.

Guo, Y. et al. (2023) ‘Thalamic network under wakefulness after sleep onset and its coupling with daytime fatigue in insomnia disorder: An EEG-fMRI study.’, Journal of affective disorders, 334, pp. 92–99. Available at: https://doi.org/10.1016/j.jad.2023.04.100.

Heeren, M. et al. (2011) ‘Cerebral glucose utilisation in hepatitis C virus infection-associated encephalopathy’, Journal of Cerebral Blood Flow & Metabolism, 31(11), pp. 2199–2208. Available at: https://doi.org/10.1038/jcbfm.2011.82.

Hesse, S. et al. (2014) ‘Altered serotonin transporter availability in patients with multiple sclerosis.’, European journal of nuclear medicine and molecular imaging, 41(5), pp. 827–835. Available at: https://doi.org/10.1007/s00259-013-2636-z.

de Lange, F.P. et al. (2004) ‘Neural correlates of the chronic fatigue syndrome--an fMRI study.’, Brain : a journal of neurology, 127(Pt 9), pp. 1948–1957. Available at: https://doi.org/10.1093/brain/awh225.

Li, J. et al. (2017) ‘Alterations in regional homogeneity of resting-state brain activity in fatigue of Parkinson’s disease.’, Journal of neural transmission (Vienna, Austria : 1996), 124(10), pp. 1187–1195. Available at: https://doi.org/10.1007/s00702-017-1748-1.

Lin, F. et al. (2019) ‘Altered nuclei-specific thalamic functional connectivity patterns in multiple sclerosis and their associations with fatigue and cognition.’, Multiple sclerosis (Houndmills, Basingstoke, England), 25(9), pp. 1243–1254. Available at: https://doi.org/10.1177/1352458518788218.

Liu, K. et al. (2016) ‘Mental fatigue after mild traumatic brain injury: a 3D-ASL perfusion study.’, Brain imaging and behavior, 10(3), pp. 857–868. Available at: https://doi.org/10.1007/s11682-015-9492-3.

Menning, S. et al. (2017) ‘Changes in brain activation in breast cancer patients depend on cognitive domain and treatment type.’, PloS one, 12(3), p. e0171724. Available at: https://doi.org/10.1371/journal.pone.0171724.

Mizuno, K. et al. (2015) ‘Impaired neural reward processing in children and adolescents with reactive attachment disorder: A pilot study.’, Asian journal of psychiatry, 17, pp. 89–93. Available at: https://doi.org/10.1016/j.ajp.2015.08.002.

Nordin, L.E. et al. (2016) ‘Post mTBI fatigue is associated with abnormal brain functional connectivity.’, Scientific reports, 6, p. 21183. Available at: https://doi.org/10.1038/srep21183.

Provenzano, D. et al. (2020) ‘Logistic Regression Algorithm Differentiates Gulf War Illness (GWI) Functional Magnetic Resonance Imaging (fMRI) Data from a Sedentary Control.’, Brain sciences, 10(5). Available at: https://doi.org/10.3390/brainsci10050319.

Shan, A. et al. (2023) ‘Aberrant voxel‐based degree centrality and functional connectivity in Parkinson’s disease patients with fatigue.’, CNS Neuroscience & Therapeutics, 29(9), pp. 2680–2689. Available at: https://doi.org/10.1111/cns.14212.

Spiteri, S. et al. (2019) ‘Neural correlates of effort-dependent and effort-independent cognitive fatigue components in patients with multiple sclerosis.’, Multiple sclerosis (Houndmills, Basingstoke, England), 25(2), pp. 256–266. Available at: https://doi.org/10.1177/1352458517743090.

Staud, R. et al. (2018) ‘Task Related Cerebral Blood Flow Changes of Patients with Chronic Fatigue Syndrome: An Arterial Spin Labeling Study.’, Fatigue : biomedicine, health & behavior, 6(2), pp. 63–79. Available at: https://doi.org/10.1080/21641846.2018.1453919.

Svolgaard, O. et al. (2018) ‘Cerebellar and premotor activity during a non-fatiguing grip task reflects motor fatigue in relapsing-remitting multiple sclerosis.’, PloS one, 13(10), p. e0201162. Available at: https://doi.org/10.1371/journal.pone.0201162.

Svolgaard, O. et al. (2022) ‘Mapping grip-force related brain activity after a fatiguing motor task in multiple sclerosis.’, NeuroImage. Clinical, 36, p. 103147. Available at: https://doi.org/10.1016/j.nicl.2022.103147.

Wagner, B. et al. (2022) ‘Is There Reduced Hemodynamic Brain Activation in Multiple Sclerosis Even with Undisturbed Cognition?’, International journal of molecular sciences, 24(1). Available at: https://doi.org/10.3390/ijms24010112.

Wang, Z. et al. (2021) ‘Single mild traumatic brain injury deteriorates progressive interhemispheric functional and structural connectivity.’, Journal of Neurotrauma, 38(4), pp. 464–473. Available at: https://doi.org/10.1089/neu.2018.6196.

Washington, S.D. et al. (2020a) ‘Exercise alters cerebellar and cortical activity related to working memory in phenotypes of Gulf War Illness.’, Brain communications, 2(1), p. fcz039. Available at: https://doi.org/10.1093/braincomms/fcz039.

Washington, S.D. et al. (2020b) ‘Exercise alters brain activation in Gulf War Illness and Myalgic Encephalomyelitis/Chronic Fatigue Syndrome.’, Brain communications, 2(2), p. fcaa070. Available at: https://doi.org/10.1093/braincomms/fcaa070.

Wortinger, L.A. et al. (2016) ‘Aberrant Resting-State Functional Connectivity in the Salience Network of Adolescent Chronic Fatigue Syndrome.’, PloS one, 11(7), p. e0159351. Available at: https://doi.org/10.1371/journal.pone.0159351.

Wortinger, L.A. et al. (2017a) ‘Emotional conflict processing in adolescent chronic fatigue syndrome: A pilot study using functional magnetic resonance imaging.’, Journal of Clinical and Experimental Neuropsychology, 39(4), pp. 355–368. Available at: https://doi.org/10.1080/13803395.2016.1230180.

Wortinger, L.A. et al. (2017b) ‘Altered right anterior insular connectivity and loss of associated functions in adolescent chronic fatigue syndrome’, PLOS ONE, 12(9), p. e0184325. Available at: https://doi.org/10.1371/journal.pone.0184325.

Wu, L. et al. (2016) ‘Altered intra- and interregional synchronization in relapsing-remitting multiple sclerosis: a resting-state fMRI study.’, Neuropsychiatric disease and treatment, 12, pp. 853–862. Available at: https://doi.org/10.2147/NDT.S98962.

Zhang, J. et al. (2017) ‘Abnormal resting‐state neural activity and connectivity of fatigue in parkinson’s disease.’, CNS Neuroscience & Therapeutics, 23(3), pp. 241–247. Available at: https://doi.org/10.1111/cns.12666.

Zhao, Y. et al. (2024) ‘Brain abnormalities in survivors of COVID-19 after 2-year recovery: a functional MRI study.’, The Lancet regional health. Western Pacific, 47, p. 101086. Available at: https://doi.org/10.1016/j.lanwpc.2024.101086.

Zhou, F. et al. (2016) ‘Resting State Brain Entropy Alterations in Relapsing Remitting Multiple Sclerosis.’, PloS one, 11(1), p. e0146080. Available at: https://doi.org/10.1371/journal.pone.0146080.

Zunini, R.A.L. et al. (2013) ‘Differences in verbal memory retrieval in breast cancer chemotherapy patients compared to healthy controls: A prospective fMRI study.’, Brain Imaging and Behavior, 7(4), pp. 460–477. Available at: https://doi.org/10.1007/s11682-012-9213-0.
